# Subdural Hemorrhage Due to Acquired Von Willebrand Syndrome in a Patient With Polycythemia Vera

**DOI:** 10.7759/cureus.16625

**Published:** 2021-07-25

**Authors:** Khalid Shalaby, Jussie Correia Lima, Robert Szulawski

**Affiliations:** 1 Internal Medicine, University of Connecticut Health, Farmington, USA; 2 Neurology, Hartford Hospital, Hartford, USA; 3 Internal Medicine, Hartford Hospital, Hartford, USA

**Keywords:** von willebrand diseases, myeloproliferative disorder, intracranial hemorrhage, chinese herbal drugs, platelet function test, ddavp

## Abstract

Polycythemia vera (PV) is a chronic myeloproliferative neoplasm associated with thrombosis. A 48-year-old female with PV presented with right eye pain following a low-impact head trauma. She consumed aspirin for analgesia and took preparations of Chinese herbs. CT head revealed right-sided subdural hematoma. She had reduced Von Willebrand activity to 26%. Direct angiographic imaging showed an aneurysm arising from a right middle cerebral atery (MCA) branch. The patient was given 1-deamino-8-D-arginine vasopressin (DDAVP) prior to the craniotomy. Intra-operative examination revealed that the aneurysm-like structure was a small grape-like structure of the fibrinous part of the subdural membrane that had formed from the subdural hematoma. Acquired von Willebrand syndrome (AVWS) is an important risk factor for bleeding in PV. DDAVP may be useful to increase levels of Von Willebrand Factor (VWF) and decrease the risk of bleeding perioperatively. Exogenous substances such as ginseng should be investigated as possible contributors to bleeding tendency and discontinued.

## Introduction

Polycythemia vera (PV) is a chronic myeloproliferative neoplasm (MPN) characterized by an increase in hematocrit, RBC count, and hypercellularity of bone marrow [1]. PV affects one to two individuals per 100,000 person-years in the United States [2]. Symptoms include fevers, night sweats, weight loss, pruritis as well as microvascular complications manifesting as headache, dizziness, and numbness [1]. Macro-thrombosis is one of the most common major complications of PV and occurs in 34%-39% of patients at the time of diagnosis [3]. Patients with low-risk PV (<60 years old and no history of cardiovascular events) are treated with serial phlebotomy with a target hematocrit <45% and low dose aspirin for primary prophylaxis of thrombotic events [4,5]. Paradoxically, patients with PV may also have inhibited hemostasis and a high degree of clinical suspicion is needed to prevent potentially life-threatening complications. Here, we report a case of intracranial bleeding in PV due to acquired Von Willebrand syndrome (AVWS) with concomitant acquired platelet dysfunction due to aspirin and herbal remedies.

## Case presentation

A 48-year-old female with a past medical history of low-risk JAK2 positive PV presented to the emergency department with a chief complaint of right eye pain for eight days. Her pain started after she had an accidental low-impact trauma inflicted by her child. She described non-pulsatile, dull pain with increasing intensity overlying her right eye. She had no visual changes in her right eye and unchanged chronic left eye myopia. To attenuate the pain, the patient consumed 12 tablets of 81 mg aspirin every day for three days at the onset of pain. She took preparations of ginseng and other unspecified Chinese herbs and prepared a herbal poultice to place over her right eye.

Her PV was diagnosed in China more than 15 years ago. She was compliant with therapeutic phlebotomies every two months for treatment of her PV, however recently she was not able to get to the clinic due to novel coronavirus restrictions. She was treated in the past with Hydroxyurea but was discontinued due to severe mucositis. Three years before presentation to our institution, she had been having heavy menstrual bleeding. Ristocetin-induced platelet aggregation assay showed decreased Von Willebrand factor (VWF) activity. Therefore, scheduled 81 mg of aspirin daily was stopped. Abdominal ultrasound, performed for the indication of abdominal swelling, revealed splenomegaly with 19.4 cm spleen in maximal dimension as well as a fully distinct 2 cm splenule.

At the time of presentation to our institution, she had no skin or eyelid wounds, mucosal bleeding, menorrhagia, joint swelling, rash, bruises, or neurological deficits. She has no family history of coagulopathy. Her vital signs were within normal limits. Her neurological exam revealed normal extraocular muscle movement, normal visual fields, decreased left eye visual acuity that remains unchanged from previous encounters. The rest of her neurological examination was unremarkable.

**Figure 1:**
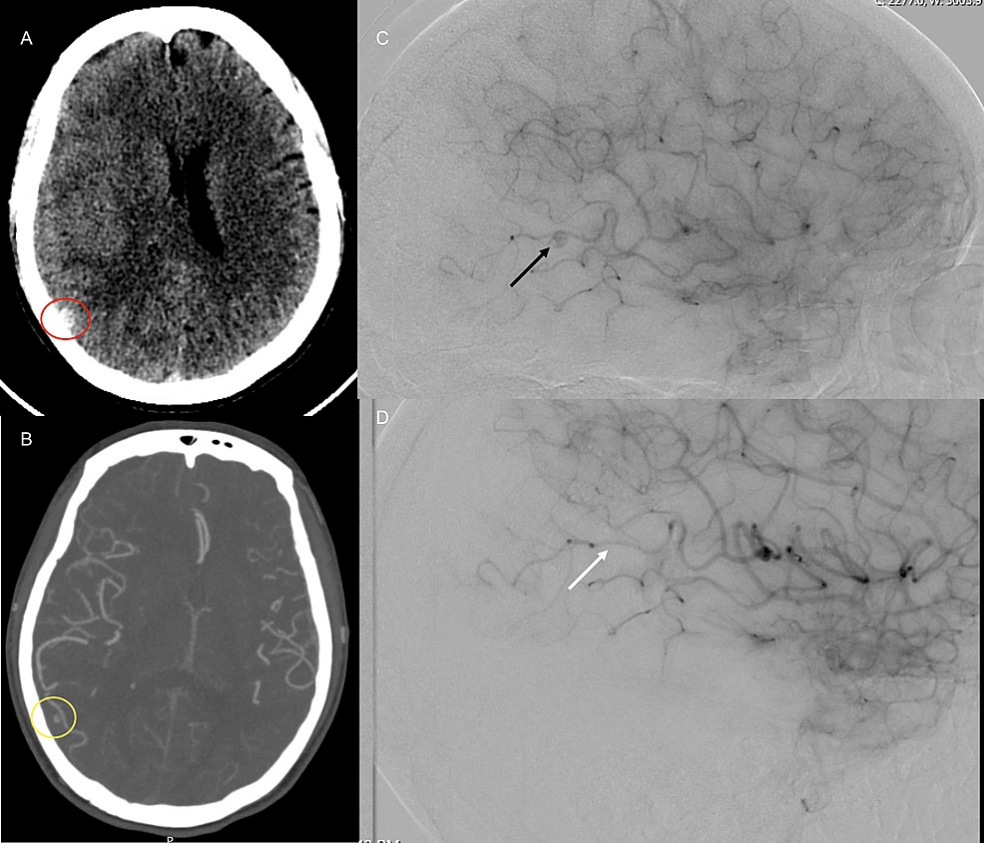
Diagnostic imaging of the head (A) CT head without contrast: Red circle - Right-sided subdural hematoma with mild mass effect. (B) CTA head: Yellow circle - Aneurysm within the right parieto-occipital region. (C) Diagnostic cerebral angiogram: Black arrow - Inferolaterally projecting aneurysm arising from the mid aspect of an M4 right middle cerebral artery branch, specifically the angular artery measuring 3.6 x 3.9 mm. (D) Diagnostic cerebral angiogram (post-surgery): White Arrow – Aneurysm is no longer visualized status post craniotomy and evacuation of subdural hematoma.

CT head without contrast was significant for right-sided subdural hematoma with a 6 mm right-to-left subfalcine shift and mass effect noted in the right ventricle without evidence of hydrocephalus (Figure 1A). CTA scan of the head and neck showed a 4-mm cortically based vascular malformation or suspected aneurysm observed within the right parieto-occipital region (Figure 1B). At the time of admission, her hemoglobin was 14.8 gm/dL, hematocrit was 53.6%, mean corpuscular volume was 68 fL, platelet count was 1,029,000/µL, and white blood cell count was 24,800/µL. Peripheral blood smear showed microcytosis, hypochromia, and polychromasia. Prothrombin time/international normalized ratio (PT/INR) and partial thromboplastin time (PTT) were within normal laboratory limits. VWF activity was reduced to 26%. She had a normal factor VIII assay. Platelet mapping revealed no inhibition to arachidonic acid (AA), but significant inhibition to adenosine diphosphate (ADP) (60% inhibited) was observed with a significant reduction in the mean amplitude of clot.

Magnetic resonance angiography (MRA) revealed a possible saccular aneurysm, measuring up to 0.7 cm, along the lateral aspect of the right parietal lobe associated with a distal branch of the right middle cerebral artery (MCA). Apparent clotted blood material surrounding this aneurysm was observed. Direct angiographic imaging of the right internal carotid artery was then performed which showed an inferolaterally projecting aneurysm arising from the mid aspect of M4 right MCA branch, specifically the angular artery. The aneurysm in question measured 3.6 x 3.9 mm. The neck of the aneurysm was relatively wide, measuring 3.2 mm (Figure 1C). The right anterior and remaining right middle cerebral artery branches were normal in appearance without significant stenosis or major branch vessel occlusion.

Neurosurgical intervention was recommended as the distal position of the aneurysm made endovascular access difficult and the wide-necked morphology of the aneurysm was not amenable to vascular intervention. The patient was given 1-deamino-8-D-arginine vasopressin (DDAVP) prior to the surgery to reduce the risk of bleeding. She underwent right craniotomy and intra-op examination revealed that directly underneath the dura, there was a very thick membrane of fibrinous material as well as hemorrhage. There was a large grape-like membrane that was attached to the pia-arachnoid. There was some bleeding noted from one of the pial arterial branches on the surface. This thickened grape-like membrane was then detached from the underside of the dura and sent off to pathology. Intraoperative microscopic evaluation of the angular artery of the middle cerebral artery revealed no evidence of an aneurysm. This aneurysm-like structure was in fact a small grape-like structure of the fibrinous part of the subdural membrane that had formed from the subdural hematoma that was being fed by a pial branch of the middle cerebral artery through a parasitized process. The pathological examination of the excised specimen was consistent with a subdural membrane showing organized hematoma with highly vascularized granulation tissue. In the post craniotomy diagnostic cerebral angiogram, the aneurysm was no longer visualized. (Figure 1D). The patient recovered well postoperatively with no clinical evidence of rebleeding. She was discharged home one day later.

## Discussion

While the most common complication of PV is thrombotic events, patients with PV can rarely present with sequelae of bleeding. AVWS is an important risk factor for bleeding in PV patients [6,7] and occurs more commonly in patients with a platelets count of more than one million/dL [8]. In instances of abnormal bleeding with MPNs, it is advisable to test for disseminated intravascular coagulopathy and AVWS with VWF antigen and activity in acutely ill patients [8]. AVWS in MPN primarily results from direct adsorption of high molecular weight multimers of VWF to blood cells, in particular platelets [9]. Cytoreductive measures are reported to reduce bleeding and result in remission of AVWS [10]. Severe thrombocytosis (>1.5 million/dL) may warrant plateletpheresis [8].

DDAVP may be useful to increase levels of VWF and decrease the risk of bleeding perioperatively and in life-threatening bleeding incidents [9]. One caveat to the use of DDAVP is that it is not always effective in the treatment of AVWS. This is attributed to the presence of immunoglobulin inhibitors of VWF, tachyphylaxis, or endothelial stores depletion [10,11]. In this case, we gave DDAVP to decrease the risk for bleeding during the surgery and the risk for recurrence of the subdural hematoma. If bleeding persists despite treatment with DDAVP, high dose factor VIII concentrate and intravenous immunoglobulins have been reported to be useful in controlling bleeding [9].

Exogenous substances should also be investigated as possible contributors to bleeding tendency and discontinued [9]. It is curious in this case that platelet mapping did not reveal any inhibition to AA despite the last aspirin use approximately five days before presentation. Marked attenuation of the ADP effect was found. We suspect that hypersplenism dramatically reduced the effective half-life of the aspirin-inhibited platelets leading to this unexpected effect [12]. Chinese herbs, including ginseng, are known to exert significant antiplatelet effects [13], and this was responsible for the potent attenuation of the ADP effect as the patient had continued taking herbals until presentation.

## Conclusions

While many cases of bleeding in MPN0associated AVWS are minor, this case illustrates that major complications of AVWS can manifest after minor trauma. Clinicians should always be aware of the potential for major bleeding tendency, especially when multiple pathways in hemostasis are suppressed. Cytoreduction is crucial in the prevention of AVWS in PV and essential thrombocythemia. DDAVP is used in the treatment of AVWS preoperatively and in life-threatening bleeding. Plateletpheresis, intravenous immunoglobulins, and factor VIII concentrate are important lines of therapy when DDAVP is ineffective.
